# Damage-mediated macrophage polarization in sterile inflammation

**DOI:** 10.3389/fimmu.2023.1169560

**Published:** 2023-07-03

**Authors:** Gábor Koncz, Viktória Jenei, Márta Tóth, Eszter Váradi, Balázs Kardos, Attila Bácsi, Anett Mázló

**Affiliations:** ^1^ Department of Immunology, Faculty of Medicine, University of Debrecen, Debrecen, Hungary; ^2^ ELKH-DE Allergology Research Group, Debrecen, Hungary; ^3^ Institute of Genetics, Biological Research Centre, Eotvos Lorand Research Network, Szeged, Hungary; ^4^ Doctoral School in Biology, University of Szeged, Szeged, Hungary

**Keywords:** macrophage, polarization, differentiation, sterile inflammation, DAMP, RAMP, SPM

## Abstract

Most of the leading causes of death, such as cardiovascular diseases, cancer, dementia, neurodegenerative diseases, and many more, are associated with sterile inflammation, either as a cause or a consequence of these conditions. The ability to control the progression of inflammation toward tissue resolution before it becomes chronic holds significant clinical potential. During sterile inflammation, the initiation of inflammation occurs through damage-associated molecular patterns (DAMPs) in the absence of pathogen-associated molecules. Macrophages, which are primarily localized in the tissue, play a pivotal role in sensing DAMPs. Furthermore, macrophages can also detect and respond to resolution-associated molecular patterns (RAMPs) and specific pro-resolving mediators (SPMs) during sterile inflammation. Macrophages, being highly adaptable cells, are particularly influenced by changes in the microenvironment. In response to the tissue environment, monocytes, pro-inflammatory macrophages, and pro-resolution macrophages can modulate their differentiation state. Ultimately, DAMP and RAMP-primed macrophages, depending on the predominant subpopulation, regulate the balance between inflammatory and resolving processes. While sterile injury and pathogen-induced reactions may have distinct effects on macrophages, most studies have focused on macrophage responses induced by pathogens. In this review, which emphasizes available human data, we illustrate how macrophages sense these mediators by examining the expression of receptors for DAMPs, RAMPs, and SPMs. We also delve into the signaling pathways induced by DAMPs, RAMPs, and SPMs, which primarily contribute to the regulation of macrophage differentiation from a pro-inflammatory to a pro-resolution phenotype. Understanding the regulatory mechanisms behind the transition between macrophage subtypes can offer insights into manipulating the transition from inflammation to resolution in sterile inflammatory diseases.

## Graphical Abstract

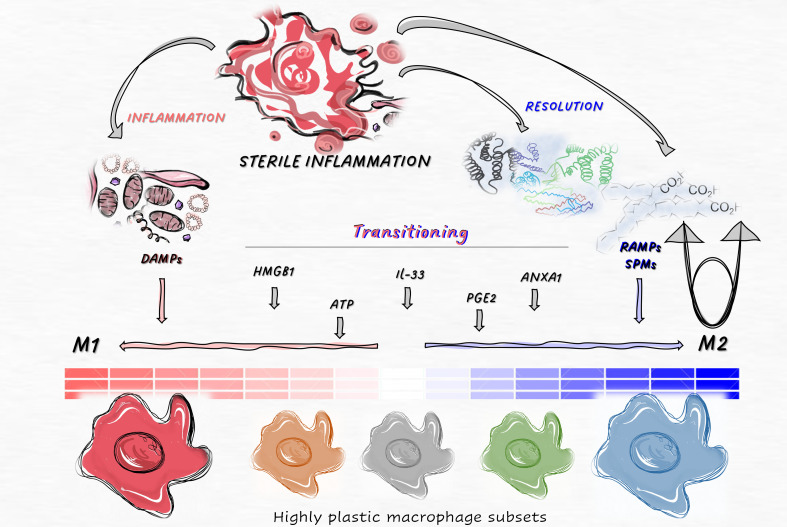

## 1 Introduction

Diseases associated with inflammation are major contributors to global mortality. Conditions such as cardiovascular and neurodegenerative diseases, cancer, various lung, kidney, and liver diseases, as well as autoimmune diseases, are all connected to sterile inflammation (1, 2). The social impact of these diseases and sterile inflammation surpasses that of infectious diseases. The inflammatory response is initiated by tissue-resident cells, leading to the infiltration of various cells and substances from the bloodstream. This complex immune response triggers the production of inflammatory mediators, resulting in tissue damage. However, over time, the inflammatory process transitions towards tissue regeneration, facilitated by the release of immunosuppressive cytokines, specialized pro-resolving mediators (SPMs), and growth factors (3, 4).

For decades, it has been known that sentinel cells of the immune system are capable of detecting and responding to various danger signals, not limited to those derived from pathogens. Consequently, both pathogen-associated molecular patterns (PAMPs) and damage-associated molecules (DAMPs) can activate tissue-resident cells. While the immune response to pathogenic invaders is primarily focused on eliminating microbes, sterile inflammation aims to resolve inflammation instead of removing the pathogen (5). Due to these distinct objectives, inflammatory responses induced solely by DAMPs in sterile conditions and those associated with pathogen-induced PAMPs may differ more than anticipated. Specifically, the kinetics and ratio of inflammation-to-resolution processes can significantly vary between sterile and pathogen-induced inflammation (6).

While sterile inflammation is typically dependent on damage-associated molecular patterns (DAMPs), the primary source of DAMPs is the release of intracellular components during cell death processes. Consequently, the initial stage of sterile inflammation often involves the activation of uncontrolled necrotic cell death or the newly described regulated necrotic cell death processes (7). During cell death, resolution-associated molecules (RAMPs) are simultaneously produced alongside DAMPs, thereby enhancing anti-inflammatory processes (4, 8). These regulated necrotic cell death pathways, which vary in terms of membrane permeabilization kinetics and mechanisms, secrete distinct combinations of RAMPs and DAMPs, as we have previously outlined (1). Since the ratio of these molecules influences tissue regeneration and can also trigger various innate and adaptive immune responses, the unique profile of these released mediators elicits diverse resolution and immune-related reactions depending on each specific cell death pathway (9).

Tissue-specific factors, such as the general regenerative capacity, immune and stem cell composition of the given tissue, the effects of the neuroendocrine system, etc. all influence the steps of the inflammatory and regenerative processes. Certain stimuli that trigger sterile inflammation, such as burn (10), cancer (11), trauma (12), autoimmunity (13), toxins, ischemia reperfusion injury and crystals (2), can induce different cell compositions and responses at the site of insults. However, according to currently accepted theory, the turning point from inflammatory responses to resolution depends on the macrophage population transitioning from a pro-inflammatory to a pro-resolution phenotype in each unique tissue environment (14).

Macrophages play crucial roles as primary responders, phagocytes, and professional antigen-presenting cells in the afferent, central, and efferent phases of immune responses. There are two distinct functional extremes within macrophage subtypes: the classical proinflammatory and the alternative pro-resolution subpopulations. In addition to these extremes, macrophage subsets with high plasticity exhibit functional and phenotypic differences (15). These different macrophages can function sequentially depending on the phase of inflammation, but they can also act concurrently. The relative ratio of these subtypes determines the balance between inflammation and resolution, ultimately affecting the homeostatic or pathological outcome.

Pathological sterile inflammation can arise from abnormal modes or intensities of cell death (9, 16), the dysregulated release of damage-associated molecular patterns (DAMPs) (as discussed in our review articles (1, 9)), or failure in transitioning from inflammation to resolution (17). In this review, we aim to provide a summary of how RAMPs and DAMPs - released during sterile tissue damage - influence the differentiation of monocytes into macrophages. Additionally, we examine how these factors regulate the function of inflammatory and tolerogenic macrophage subsets.

## 2 DAMPs, RAMPs and SPMs: regulators of sterile inflammation

In the absence of pathogens, sterile inflammation is triggered by the release of damage-associated molecular patterns (DAMPs) associated with tissue damage (**Table 1**). DAMPs can originate from intracellular molecules released during necrotic processes, as well as from the extracellular matrix damage or the secretion of intracellular molecules through vesicles in response to cellular stress signals (22). Accordingly, modified matrix proteins, the extracellular appearance of cytosolic, nuclear, mitochondria-, ER-derived molecules, or vesicle-released proteins can activate the sentinel cells of innate immunity, resulting in the production of inflammatory mediators (23).

**Table 1:** DAMPs, RAMPs and SPMs as coordinators of necro-resolution and necro-inflammation.

	Polarizing patterns	Producing/releasing cell	Human cell-derived mediators	Effect	Ref.
**Necro-inflammation-inducing**	DAMP	Dying cell	DNA, IL-1α/β, Histones, S100 proteins, HSPs, Uric acid, EMAPII, eCRIP, Cyclophilin A, SAP130, ASC specks, Oxidized phospholipids, Malondialdehyde	Induction of inflammation	(9, 18)
**Necro-resolution-inducing**	RAMP	Dying cell, tissue-resident cells	HSP10, HSP27, αBC, BiP, PS	Help to counterbalance the inflammatory effects of PAMPs and DAMPs	(8)
	SPM	Efferocytes, tissue resident mesenchymal stromal cells	Lipoxins, Resolvins, Protectins, Maresins	Increasing the non-inflammatory phagocytosis of apoptotic PMNs - limit neutrophil tissue accumulation, counter-regulate pro-inflammatory cytokines and encourage macrophage phagocytosis	(19–21)
**Transition from inflammation to resolution**	DAMP and RAMP	Dying cell	HMGB1, ATP, IL-33, PGE2, AnnexinA1	Inflammatory molecules, but under certain conditions, induce signals for resolution	(1)

**Figure 1:**
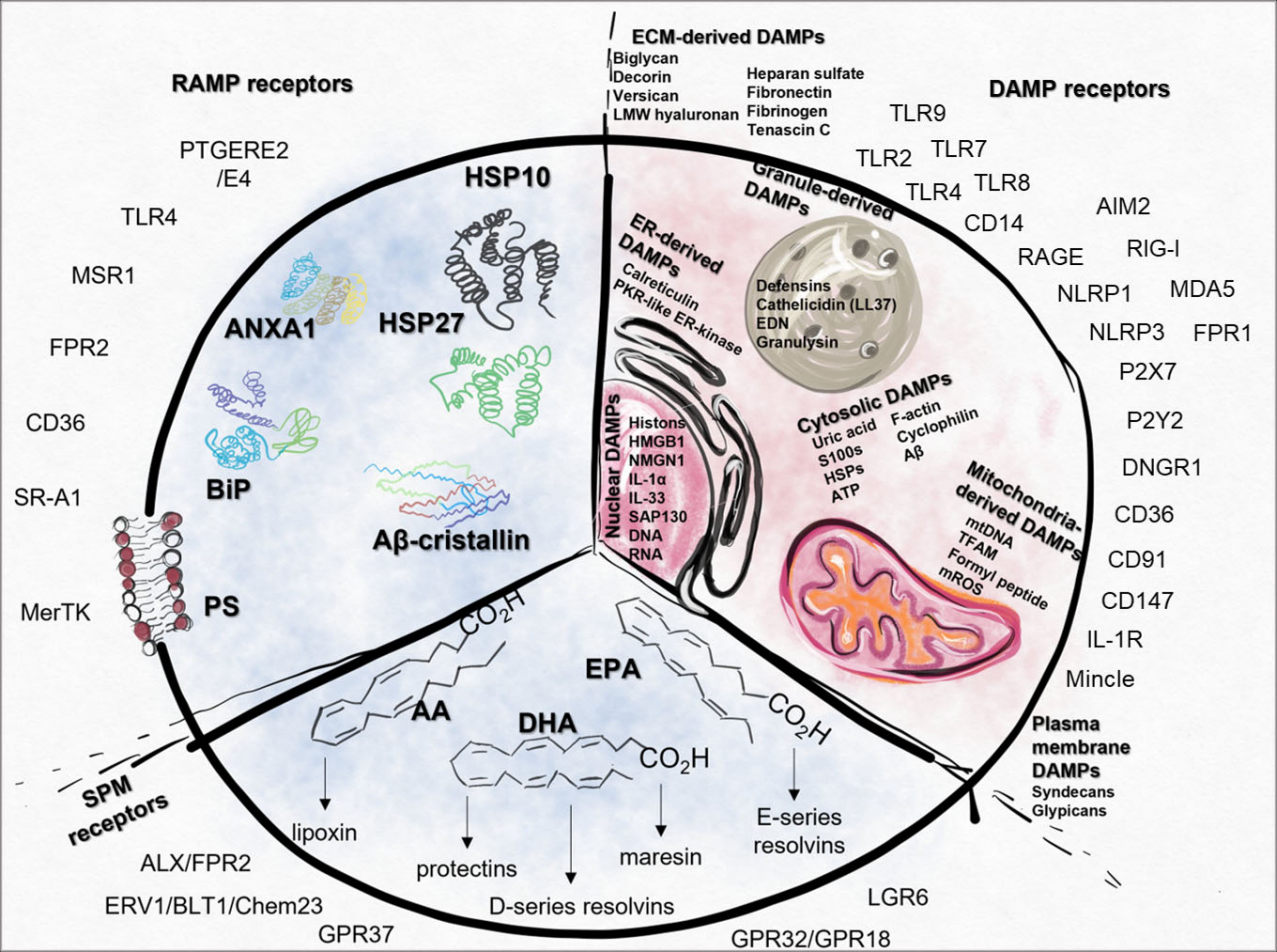
Summary of DAMPs, RAMPs, SPMs and their receptors. Intracellular (IC) DAMPs can be released from the cells during cell death or cellular stress and the damage of ECM also results in the generation of DAMPs (EC). DAMP-sensing receptors are responsible for the recognition of ECM, cytosolic, nuclear, mitochondria-, ER-, plasma membrane- or granule-derived DAMPs. In case of cell damage, RAMPs can also be released, therefore their receptors act simultaneously with the signals generated by DAMPs. SPMs can be produced from AA, DHA or EPA, which directs the resolution process by activating different SPM receptors. AA, arachidonic acid; AIM2, absent in melanoma 2; ALX, Lipoxin receptor; ANXA1, Annexin A1; Aβ, amyloid beta; BiPS, Binding immunoglobulin protein; CD, cluster of differentiation; CD14, cluster of differentiation 14; Chem23, Chemokine like receptor 23; CLEC4E, C-Type; Lectin Domain Family 4 Member E, CREB, Cyclic AMP-Responsive Element-Binding Protein; DAMP, danger associated molecular pattern; DHA, docosapentaenoic acid; ECM,, extracellular matrix; EPA, eicosapentaenoic acid; ERV1, human resolvin E1 receptor; FPR, Formyl peptide receptor; GPR37, G Protein-Coupled Receptor 37; HMGB1, High mobility group box 1 protein; HSP10, Heat shock protein 10; IC, intracellular; LGR6, Leucine-rich repeat-containing G protein-coupled receptor 6; LMW, Low-molecular-weight; LPS, Lipopolysaccharides; MDA5, melanoma differentiation-associated protein 5; MerTK, MER proto-oncogene, tyrosine kinase; Mincle, Macrophage Inducible C-Type Lectin; mtDNA, Mitochondrial DNA; MRC-1, Mannose-Receptor C type 1; MSR1, Macrophage scavenger receptor 1; NLRP1, NLR family pyrin domain containing 1; NLRP3, NLR family pyrin domain containing 3; PS, Phosphatidylserine; PTGERE2,, Prostaglandin E2 receptor; RAGE, Receptor for Advanced Glycation Endproducts; RAMP, resolution-associated molecular patterns; RIG-I, retinoic acid-inducible gene I; SAP130, Histone deacetylase complex subunit; SPM, Specialized pro-resolving mediators; SR-A1, Class, A1 scavenger receptors; ST2, Interleukin-1 receptor-like 1; TLR, Toll-like receptor; TREM1/2, Triggering receptor expressed on myeloid cells 1/2.

Other dying cell-derived factors, RAMPs, and SPMs, such as protectins, resolvins, lipoxins and maresins, may also influence the inflammatory processes and induce necro-resolution. Immune cells – such as macrophages – sense the presence of dead cells, and engulf them, which activates immunosuppressive and tissue regenerative functions in a time-dependent manner by inducing the production of anti-inflammatory cytokines and further SPMs. The relative ratio of DAMPs/RAMPs and pro/anti-inflammatory mediators contribute to the formation of a polarizing microenvironment (**Figure 1**).

Importantly, the tipping point, when and how the inflammatory reactions turn into a resolution process, is poorly understood. Certain DAMPs, which originally trigger proinflammatory processes, can be converted into forms that trigger anti-inflammatory processes by further modifications, such as the oxidation of HMGB1 or the processing of ATP into adenosine. Certain receptors of dying cells (such as the “don’t eat me” signals CD24, CD31, and CD47) or soluble molecules that can bind to DAMPs (C1q, CD52) and stimulate the inhibitory receptors of efferocytes, thus transforming DAMP-mediated signaling into anti-inflammatory ones. Tissue damage increases the amount and proportion of dead cells. Phosphatidylserine (PS)-expressing cell corps activate MerTK-induced signaling in efferocytes, leading to SPM and anti-inflammatory cytokine production and polarization of macrophages to an M2 phenotype. The effects of additional DAMPs such as IL-33, Annexin A1 (ANXA1), and certain inflammatory mediators (PGE2) depend on the cells and/or receptors that sense them. Thus, alterations in the tissue environment can change their effect into an anti-inflammatory effect [reviewed in (1)].

## 3 Overview of M1 and M2 macrophage differentiation

The immune response is influenced not only by the types of RAMPs and DAMPs released but also by the cells responsible for sensing them. It is worth noting that the same RAMP or DAMP molecules can elicit different responses depending on the activation of either tolerogenic or inflammatory subpopulations of tissue-resident cells. Simultaneously, the recognition of these factors can impact the differentiation of monocytes, the polarization and the functional activity of macrophages, allowing them to finely adjust their activity and adapt to the current environment (24).

Macrophages that are in a resting state are referred to as inactive, non-polarized “M0” type macrophages. The M0 macrophages represent a diverse population characterized by their origin and tissue-specific functions. In this context, our focus is on the overall immune-related functions of macrophages, while a comprehensive summary of their specific characteristics can be found in reviews published elsewhere (3, 25, 26).

**Figure 2:**
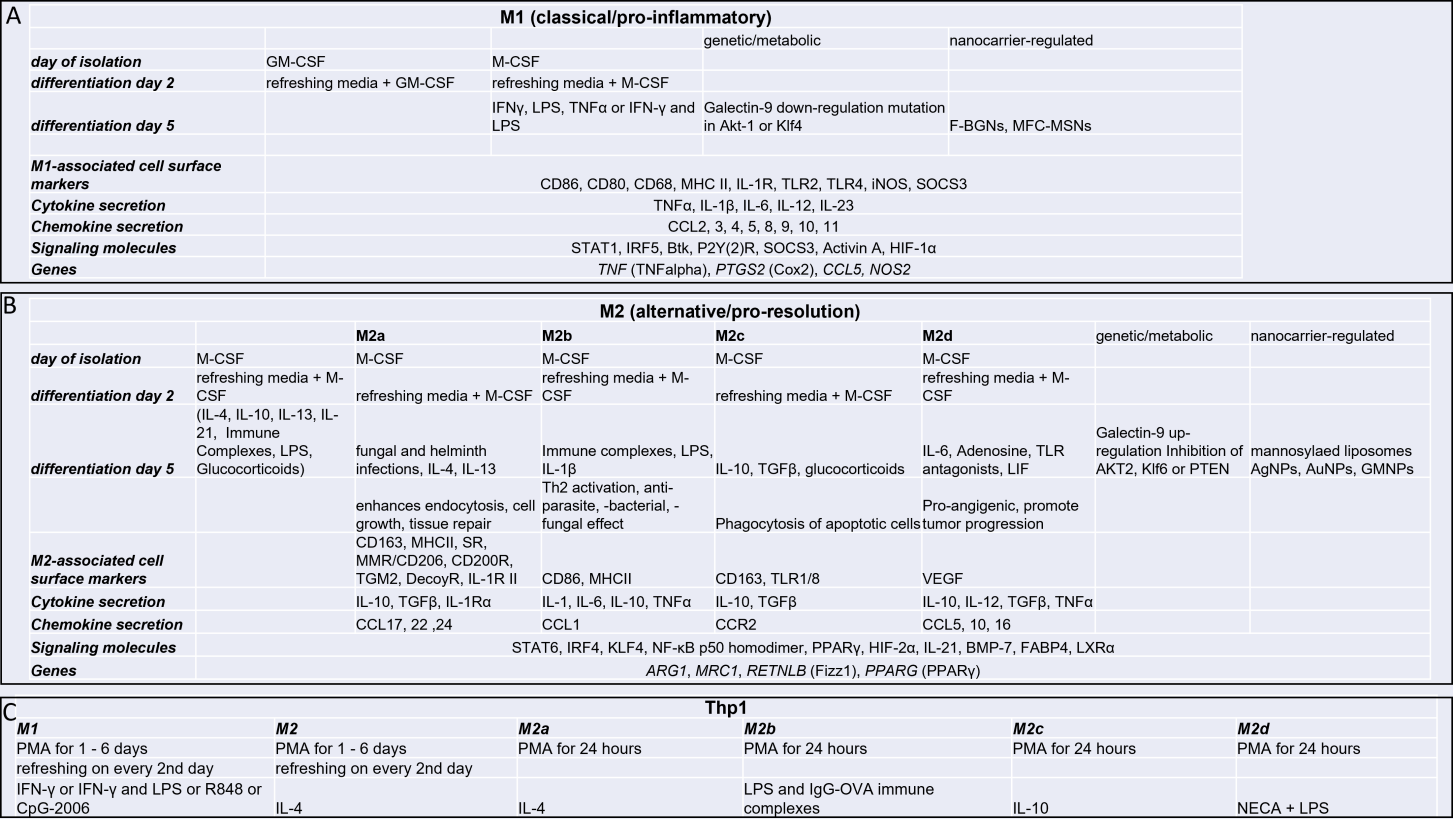
Main characteristics of in vitro differentiated and polarized M1 and M2 macrophages. Each phenotype could be formed in response to certain inductors: M1 upon LPS and/or IFN-γ **(A)**; М2а in the presence of IL4/IL13; M2b in response to immune complexes; M2c could be generated after binding of IL-10 or TGF-β snd M2d with IL6 and adenosine **(B)**; The THP1 cell line is also often used for macrophage differentiation **(C)**. Arg1- Arginase-1, BMP, Bone morphogenetic protein; CCL2, Monocyte chemoattractant protein-1; COX-2, cyclooxygenase-2; COX-2, cyclooxygenase-2; FABP4, Fatty acid binding protein 4; Fizz1, Resistin-like molecule alpha1; GM-CSF, Granulocyte-macrophage colony-stimulating factor; HIF-1-α, Hypoxia-Inducible Factor Promotes; IFN-γ, Interferon gamma; IL-1R, Interleukin-1 receptor; iNOS, Inducible nitric oxide synthase; iNOS, Inducible nitric oxide synthase; Klf4, Krüppel-like factor 4; M-CSF, macrophage colony-stimulating factor; MRC-1, Mannose-Receptor C type 1; PMA, Phorbol myristate acetate; PPARs, peroxisome proliferator-activated receptors; PPARs, peroxisome proliferator-activated receptors; PTEN, lipid and protein phosphatase and tensin homolog; SOCS3, suppressor of cytokine signaling-3; STAT1, Signal transducer and activator of transcription 1; TGF-beta, Transforming growth factor-beta; TNF, Tumor necrosis factor; VEGF, Vascular endothelial growth factor.

Functionally, classical macrophage-like cells (M1) exhibit pro-inflammatory, anti-microbial, and tumor-resistant properties. They are characterized by increased expression of CD80, CD86, and the production of immunostimulatory cytokines (TNFα, IL-1β, IL-6, IL-12, IL-23). M1 cells also secrete a wide range of chemokines (**Figure 2** panel A), which facilitate the activation and proliferation of NK cells, CD4^+^ Th1 cells, and CD8^+^ T-lymphocytes.

On the other hand, alternative macrophage-like cells (M2 cells) play a crucial role in anti-inflammatory and resolution processes. They are predominantly identified by the presence of CD163 and CD206 markers. M2 cells possess the ability to produce both inflammatory (IL-6, TNFα, IL-12) and anti-inflammatory (IL-10, TGFβ) cytokines, contributing to the restoration of homeostasis (**Figure 2** panel B). Their functions as immunoregulators and promoters of tumor growth. M2 cells exhibit high phagocytic capacity and are involved in producing extracellular matrix (ECM) components, angiogenic factors, chemotactic factors, and various cytokines. In addition to their role in pathogen defense, they also participate in the clearance of apoptotic cells, suppression of inflammatory responses, and promotion of wound healing and tissue remodeling.

Within the two endpoints, between cells M1 and M2, several macrophage subpopulations can be created *in vitro* or identified in different tissues under steady state or different pathological conditions. M2 macrophages were further divided into M2a, M2b, M2c, and M2d subtypes based on applied stimuli and induced transcriptional changes (27) (**Figure 2**).

The transition from innate M1 to M2 states indicates that macrophage differentiation and activation occur along a spectrum, where distinct epigenetic, metabolic, phenotypic, morphological, and functional characteristics are acquired by different cell populations. The collective expression of macrophage-derived factors and cell surface markers determines the functional properties of macrophages. It is important to note that individual cells can co-express “markers” associated with both M1 (e.g., CD163, TNFα) and M2 (CD209, TGFβ) states, emphasizing the complexity of polarization (28). Furthermore, there can be the simultaneous presence of functionally distinct subpopulations of macrophages, enabling precise modulation of tissue responses.

The fate of monocyte/macrophage populations may also vary in the post-inflammatory phase; some of the cells are likely to die due to the inflammatory reaction. Surviving cells can undergo phenotype conversion in situ, leading to the appearance of M2-like resident macrophages, but a few of them, trained monocytes, preserve their phenotype for a longer period, even months, “remembering” previous inflammatory stimuli that affected them (29, 30).

In this chapter, we aim to provide a general overview of the commonly used and widely accepted methods for *in vitro* macrophage differentiation and polarization. In recent decades, various terms have been used to classify different types of macrophages exposed to cytokines or Toll-like receptor (TLR) agonists. In human studies, peripheral-blood monocytes or THP-1, a human monocyte-derived cell line from a patient with acute monocytic leukemia, have been commonly utilized as an *in vitro* system for generating macrophage subpopulations. Although not entirely representative of *in vivo* cell populations, this model system has proven to be one of the most reproducible experimental standards (29). Initially, the impact of interferon-γ (IFNγ) and/or lipopolysaccharide (LPS) on M1 macrophages and interleukin-4 (IL-4) on M2 macrophages was described (31). Another widely employed approach to differentiate polarized macrophages involves using GM-CSF for M1 cells and M-CSF for M2 cells alone (32). The essential protocols for *in vitro* macrophage differentiation and polarization, along with the key characteristics of the resulting M1 and M2 cells, are summarized in **Figure 2**.

Distinct differences have been observed in the transcriptomes of these generated cells. Stimulation of macrophages with IFNγ or IL-4 exclusively activates either STAT1 or STAT6 (33). Other genetic modifications also lead to shifts in macrophage polarization. For instance, IRF5 and SOCS3 drive M1 differentiation, whereas the transcription factors IRF4 or KLF4 and certain PPARs induce M2 differentiation (34). Notably, metabolic changes occur during differentiation as well. Molecules associated with anabolic growth, such as AKT2 or PTEN, promote an M1-like expression pattern, whereas the mTOR inhibitor TSC1 facilitates M2 formation (35). M2 macrophages exhibit more pronounced arginine metabolism compared to M1 macrophages, and this disparity disappears when the Slc7a2 promoter responsible for arginine transport is deleted (36).

It is important to note that *in vitro* models are not capable of modeling the complex interactions occurring within tissues, significant emphasis should be placed on considering results derived from *in vivo* systems. Although the polarization of macrophages into M1-like or M2-like states can also be identified *in vivo*, it requires a more precise characterization. Their characterization primarily relies on detailed phenotypic and functional studies, as well as single-cell analyses.

The *in vivo* differentiation of macrophages is a much more complex process influenced by numerous factors. Their characteristics are influenced by the individual’s general health condition; age (37, 38), potential pathological conditions (39–41), and the stage, flare-up, or resolution of diseases (14). There are numerous available *in vivo* model where sterile inflammation is artificially induced, resulting in tissue damage. In the case of immune reactions triggered by PAMPs, the inflammatory agent is known. However, in models that induce sterile inflammation, the question of which DAMP released due to injury serves as the initiator of immune reactions or its potential role in regulating the immune response is often not addressed (except, e.g (42–44). among others), even though DAMPs, RAMPs, and SPMs are part of the complex tissue microenvironment.

Here we collected models represent different experimental approaches used to study sterile inflammation in various contexts, but where the potential DAMPs are not identified. Sterile inflammation could be induced by overexposure to UVB irradiation (i.e., sunburn) in the mouse plantar skin (45), polyvinylpyrrolidone (PVP) in rat model (46), as well as with phorbol 12-myristate 13-acetate (PMA) to induce acute mouse ear inflammation (47). Another possibility is the stimulation with 2,6,10,14-tetramethylpentadecane (TMPD, also known as pristane) (48). Additionally, it can be evoked with cytodex bead slurry injection subcutaneously into the back space in mouse (49), furthermore, the hepatic I/R Injury also can be induced (50). The injection of cardiotoxin into the muscle (51) can be used to trigger sterile inflammation as well as the following method can be applied: injection of mice with heparin, and the use of an atraumatic clip to interrupt the arterial and portal venous blood supply to the cephalad lobes of the liver (52). In the case of autoimmune models, the DAMPs are also neglected, however their potential presence is also determining (53).

The presence of macrophages in different conditions is still a subject of conflicting studies, but the altered functions can be attributed at least in part to the changed or divergent DAMP/RAMP/SPM sensing-receptor expression patterns.

## 4 Effect of DAMPs and RAMPs released from dying cells on macrophage differentiation

In addition to cytokines or PAMPs, macrophage polarization can also be induced by different types of endogenous damage-related molecules (DAMPs and RAMPs). Non-polarized macrophages express relevant receptors for DAMP-, RAMP-, and SPM-sensing which could influence their differentiation into proinflammatory or proresolution macrophage subtypes (54). The presence of these receptors on M0 cells shows that these cells are sensitive to DAMP, RAMP and SPM signals, indicating that these stimuli may direct macrophage differentiation.

The relevance of our question is supported by the fact that sterile inflammation is induced in many *in vivo* mouse studies (48, 55), yet the role of factors released from damaged or dying cells, such as DAMPs and RAMPs, in triggering inflammation during sterile inflammation is not widely investigated. The different phases involved in tissue damage and regeneration, which heavily rely on the innate immune system, particularly monocyte-derived macrophages. These macrophages play a dual role, first in reacting to the injury, and second by removing debris, promoting resolution of inflammation, and initiating tissue repair. At the end of the inflammation phase, the pro-inflammatory phase subsides, transitioning into a regenerative inflammation phase that leads to tissue repair. There is a dynamic nature ofmacrophage response and identifies specific molecular signatures characterizing inflammatory and repair macrophages during tissue injury and repair (14).

### 4.1 Effect of DAMPs released from dying cells on macrophage differentiation

It is well known that besides IFNγ, PAMP signals, such as LPS, are the most important stimulus for inducing M1 macrophage polarization. Since DAMPs mostly stimulate a set of receptors overlapping with PAMPs (56), we can assume that DAMPs predominantly induce M1 differentiation. Accordingly, HMGB1 facilitates the reprogramming of macrophages towards an M1-like phenotype dependent on TLR4-mediated pathways (57, 58). Recombinant HSP90 expressed in extracellular vesicles shifts the differentiation of M0 macrophages towards the M1 phenotype (59). The presence of uric acid leads to the differentiation of monocytes into inflammatory M1-like macrophages through NLRP3 activation (60). Extracellular RNA (eRNA) released from different tissues induced the differentiation of M0 macrophages towards the M1 phenotype (61). Extracellular accumulation of the stress response protein eCIRP inhibited the polarization of M2 macrophages, thereby shifting the balance of macrophage subtypes towards an inflammatory phenotype (62). However, DAMP signals do not exclusively favor the formation of proinflammatory macrophages. Factors acting together with DAMPs can modify DAMP-related functions, so their effect also depends on the tissue environment. For example, DNA released from activated neutrophils during netosis induces a rapid inflammatory response, but DNAses are also secreted, eventually ceasing the proinflammatory effect of extracellular DNA (63). High doses of S100B may also contribute to subsequent tissue regeneration (64). In the presence of immune complexes, even the highly inflammatory LPS or IL-1β signals are modulated, leading to the differentiation of the M2 macrophage subtype (65).

### 4.2 Effect of RAMPs released from dying cells on macrophage differentiation

Intracellular molecules released from dying cells that play a direct role in inducing resolution favor the differentiation of M2 macrophages over M1 cells. HSP27 secretion induces the differentiation of tolerogenic macrophages in the tumor microenvironment (8). The extracellular appearance of HSP10, HSP27, BiP and ANXA1 promotes autocrine IL-10 production driving the differentiation toward the M2 phenotype (66). SPM secretion, resolving D1/E1, lipoxin A4 and maresin1 from dying cells has been demonstrated, and efferocytes engulfing dead cells can also produce SPMs (9). The common effect of the different SPMs is the enforcement of the M2 differentiation (1). PS appearing on the surface of dead cells also increases the polarization in the M2 direction through the activation of MerTK receptors on the efferocytes (67). MerTK signaling also results in SMP production, which also enhances the differentiation of M2 cells. As MerTK and SPM expression increases in M2 cells, a complex positive feedback loop appears between MerTK, SPMs, and M2 cells in the presence of dead cell corps. An increase in the amount of dying cells or M2 cells therefore turns the inflammatory signals in the direction of resolution (1).

### 4.3 Effects of DAMP conversion to RAMPs on macrophage differentiation

Depending on their environment or post-translational modifications, some molecules can function as both DAMPs and RAMPs, such as HMGB1, ATP, IL-33, prostaglandin E2 (PGE2) and ANXA1. These molecules, originally identified as DAMPs, appear to play a critical role in regulating the transition between pro-inflammatory and pro-resolution phases (1). Accordingly, they could have an ambivalent effect on macrophage polarization. The HMGB1 molecule has three functionally distinct isoforms based on the redox state of the three cysteines; disulfide-HMGB1, fully reduced, and sulfonyl-HMGB1 variations. Disulfide-HMGB1 induces inflammatory cytokine secretion and in accordance with this functionality, polarizes macrophages in the M1 direction, with a slightly different transcriptomic profile than LPS/IFNγ signals (68). Fully reduced HMGB1 is known to induce chemotaxis and has a limited effect on macrophage polarization (68). Sulfonyl-HMGB1 has been described as inactive in inducing inflammation and new observations also suggest that sulfonyl-HMGB1 increases the M2/M1 macrophage ratio (69). Extracellular ATP, another classic DAMP molecule, is converted to immunosuppressive adenosine under the action of CD39 and CD73 ectonucleotidases. Adenosine promotes differentiation towards M2 while inhibiting M1 polarization (70). Sensing ATP by any macrophage subtype leads to the release of anti-inflammatory proteins such as ANXA1, suggesting that intact ATP may also have a potential role in resolving inflammation (71). Extracellular ATP activates P2X7R on M1 polarized macrophages, leading to the release of pro-inflammatory IL-1β *via* activation of the caspase-1/NLRP3 inflammasome (72). IL-1β is an important polarizing cytokine for M2b cells under certain circumstances (65). IL-33, which belongs to the IL-1 cytokine family, it is not secreted by the ER-Golgi pathway, therefore it is mainly released from cells that lose membrane integrity. Although it is considered an alarmin molecule (with a DAMP-like function), it strongly supports the differentiation of M2 cells, thus also contributing to resolution (73). PGE2 plays a role in early inflammatory events, but the second wave of its production typically induces an anti-inflammatory process (74). Its effect on macrophage differentiation characteristically stimulates M2 polarization, while it blocks the function of M1 macrophages (75, 76). ANXA1 and its N-terminal cleavage product Ac2-26 induce differentiation of monocytes into M2a, M2c-like cells and transformation of macrophages into M2 subtypes (77). Accordingly, extracellular ANXA1 favors tissue regeneration (78, 79), while ANXA1 KO mice were characterized by impaired tissue regeneration with a predominance of the M1 phenotype (78, 80).

Cancer-driven immune editing limits or alters macrophage responsiveness to DAMPs, an example of how DAMP-mediated macrophage polarization depends on the tissue environment. The intense growth of tumors coupled with their resistance to apoptosis induces significant necrotic cell death, thus leading to intensive DAMP release. In this environment, the M1 phenotype-promoting effect of DAMP signals is abrogated and it even supports the differentiation of M2-like cells, the mechanism of which is not always clear (81). Hypoxia-induced macrophage polarization is HMGB1-dependent, significantly increasing the number of tumor-associated macrophages with an M2-like phenotype in HMGB1-positive murine and human melanomas (82). Pancreatic ductal adenocarcinoma cells with endothelial-mesenchymal transition secrete HSP90 to induce M2 polarization of macrophages (83). eRNA promotes the polarization of M2 macrophages in patients with colorectal cancer (84). S100A4 enhances protumor macrophage polarization by the upregulation of PPAR-γ (85). IL-33 enhances M2 polarization by inducing a metabolic shift toward increased cellular oxidative phosphorylation and ultimately promotes tumor growth in a B16 melanoma model (86).

Overall, it can be concluded that despite the presence of proinflammatory DAMPs, tissue resolution can also develop in special tissue microenvironments, highlighting the environmental effect on the functioning of DAMPs.

## 5 Comparison of RAMP- and DAMP-sensing receptor expression on polarized M1 and M2 cells

Depending on the differentiation state of the cells, different signaling pathways can be activated, which control whether pro- or anti-inflammatory mediators are produced in excess. If an excess of proinflammatory or proresolution macrophage subpopulation develops in the tissue environment as a result of previous stimuli, this significantly affects the immune response to a given stimulus. The different response ability of individual subpopulations may result from the non-identical expression of receptors or the inherent difference in signaling pathways. In the next chapters, we will review how DAMP and RAMP receptors are expressed in M1 and M2 cells and compare the signaling pathways activated in these cell populations.

### 5.1 Expression of DAMP-sensing receptors on polarized M1 and M2 cells

Damage-related molecules, as a group of danger signals, are mostly recognized by the same PRRs as pathogen-related patterns. However, some receptors are specialized only for the detection of DAMPs (23). A surprisingly small number of comparative studies have examined PRR expression on M1 and M2 cells (15). Based on these studies, virtually all DAMP-sensing receptors are expressed by both M1 and M2 cells (**Table 2**).

**Table 2:** DAMP, RAMP, SPM and “transitioning” molecule-sensing receptors on non-polarized, M1 and M2 cells.

		M0	M1	M2
**DAMP**	**DNA**	RAGE (87)	RAGE (88), TLR9 (89)	RAGE (87)
	**IL-1α/β**	IL-1RI IL-1RII (90)	**↑**IL-1RI (91)	**↑**IL-1RII (91)
	**Histones**	TLR2, TRL4 (92)	TLR2, TRL4, TLR9 (89)	TLR4, TLR2 (93)
	**RNA**	TRL3 (94)	TRL3 (95), TRL7 (96) TLR8 (97)	TRL3 (95) TRL7 (98)
	**S100 proteins**	RAGE (87) TRL4 (92)	RAGE (88) TRL4 (99)	RAGE (87) TLR4 (99)
	**HSPs**	TLR2, TRL4 (92) TREM-1 (100) CD14 (101)	TLR4, TLR2 (89) TREM-1 (102) TREM-2 (103) CD14	TLR4, TLR2 (93) TREM-1 (104) **↑**TREM-2 (103) CD14
	**Uric acid**	TLR2, TRL4 (92) P2X7R (105) CD14 (101)	TLR4, TLR2 (89) P2X7R (106) CD14	TLR4, TLR2 (93) **↑**P2X7R (71) CD14
	**EMAP II**	VEGFR (107)	VEGFR (108)	**↑**VEGFR (109)
	**Ecirp**	TREM-1 (100) TRL4 (92)	TREM-1 (102) TLR4, MD2 (110)	TREM-1 (104)
	**Cyclophilin A**	CD147 (111)	CD147 (112)	CD147 (112)
	**SAP130**	TRL4 (92) MINCLE (113)	TRL4 (114) **↑**MINCLE (114)	TLR4 (99) MINCLE (114)
	**ASC specks**	P2X7R (105)	P2X7R (106)	**↑**P2X7R (71)
	**Oxidized phospholipids**	TLR2 (92)	TLR2 (89)	TLR2 (93)
**RAMP**	**HSP27**	TRL4 (92) SR-AI (115)	TRL4 (99) SR-AI (116)	TLR4 (99) SR-AI (117) CD36 (118)
	**PS**	MERTK (119)	MERTK (120)	MERTK (1)
**SPM**	**Resolvin E**	BLT1 (121) ERV1 (122)	BLT1 (121) ERV1 (122)	**↑**BLT1 (121) Cmklr1
	**Resolvin D**	ALX/FPR2 (123) DRV1/2	ALX/FPR2 (124)	ALX/FPR2 (124)
	**Lipoxin**	ALX/FPR2 (125)	ALX/FPR2 (124)	ALX/FPR2 (124)
	**Protectin**	GPR37 (126)	GPR37 (126)	GPR37 (126)
	**Maresin**	LGR6 (127)	**↑**LGR6 (127) RORa	LGR6 (127)
**Transition**	**HMGB1**	TLR2, TRL4 (92) RAGE (87) TREM-1 (100)	TLR2, TRL4, TLR9 (89) RAGE (88) TREM-1 (102)	TLR4, TLR2 (93) RAGE (87) TREM-1 (104)
	**ATP**	P2X7R (105) P2Y4 (105)	P2X7R (106) P2Y4 (105) P2Y2	**↑**P2X7R (71) P2Y2 **↑**CD39
	**IL-33**		ST2 (99)	**↑**ST2 (128)
	**PGE2**	EP2, EP4 (129)	**↑**EP2 (130) **↑**EP4 (131)	EP2 (132) EP4 (131)
	**AnxA1**	FPR2 (123)	**↑**FRP1, **↑**FPR2 (133)	FRP1, **↑**FPR2 (133)

↑ The arrow indicates that although certain receptors may be expressed in different macrophage subtypes, their expression can be further enhanced due to polarization.

Based on the literature, only the expression of Mincle (CLEC4E) (114), which recognizes bacterial glycolipids and SAP130 as a DAMP, and TLR2, which primarily recognizes bacterial lipids and various DAMPs, were markedly higher in M1 cells than in M2 macrophages. Thus, the different functional activity of pro- and anti-inflammatory macrophages presumably does not arise only from PRRs.

More dominant differences can be observed between the two macrophage subtypes in the expression of receptors, which can detect only danger signals, but not PAMPs, such as receptors for IL-1, heat shock proteins, VEGF, IL-33 and ATP (the last two are reviewed in the next chapter). Lower expression of IL-1RI, which has an inflammatory role, and higher expression of IL-1RII, a decoy receptor to block IL-1 signals has been published on M2 cells (27). The expression of the VEGF receptor, which detects extracellular cyclophilin A, was higher on M2 macrophages (109). Trigger receptors expressed on myeloid cells (TREM) are specialized to detect various DAMPs, such as extracellular HSP proteins, actin and cCIRP. TREM-1 is a well-known enhancer, while TREM-2 is a negative regulator of the inflammatory response (134). While TREM-1 expression is more dominant on M1 macrophages, TREM2 expression is the more typical feature of M2 macrophages based on literature data (103).

Examining the expression of the receptor, greater differences can be seen in the expression of receptors that sense DAMPs than those that sense PAMPs. However, further functional studies are needed to determine whether this divergence contributes to the functional differences between M1 and M2 macrophages.

### 5.2 Expression of RAMP and SPM sensing receptors on polarized M1 and M2 cells

Recognition of RAMPs and SPMs by diverse receptors (TLR4, SR-Al, CD36, PGE2R EP2/EP4, MERTK for RAMPs and ALX/FPR2, ChemR23, GPR32, GPR37, RORα, LGR6 for SPMs) are responsible for the initiation and completion of resolution process in a fine-tuned manner.

The receptors of secreted HSP proteins belonging to RAMPs, (HSP10, HSP27, BiP/HSPA5, αB-crystallin/HSPB5) are typically barely known (135). An interesting aspect of their anti-inflammatory effect is that HSPs involved in resolution can bind to the same receptors as HSPs with DAMP function, inhibiting those by competition. Hsp10 can interact with extracellular Hsp60, inhibiting inflammatory responses of this DAMP (136). Similarly, HSP27 binding may compete with LPS binding to TLR4 and oxLDL binding to SR-AI or CD36, thereby attenuating these activating signals (137). In addition, CD36 stimulation by HSP proteins resulted in PPARγ activation, indicating that high CD36 expression can convert the function of HSP proteins described as DAMPs into resolution-promoting activity (138). The expression of PS-recognizing MerTK receptors, which direct the efferocytosis of dead cells, is increased on M2 cells (67). As MerTK receptor signaling promotes the differentiation of macrophages to the M2 phenotype, this creates a positive feedback loop between efferocytosis and M2 differentiation (1, 139–141).

The receptors of individual SPMs are differentially expressed in M1 and M2 macrophages. Protectin and maresin receptors GPR37 and RORα are expressed at a higher level on M2 cells (126, 142), and the expression of the resolvin D and lipoxin-recognizing receptors FPR2 is also higher in THP-derived M2 than in M1 cells (133). In contrast, the expression of ChemR23 (resolvin E) and LGR6 (maresin) is higher on M1 cells (122, 127). The receptor for Resolvin D1, GPR32 is present on both LPS-activated M1 and IL-4-activated M2 macrophages, but its expression is reduced by TGFβ and IL-6 treatment (143).

We must underscore that SPMs even acting on M1 cells induce the differentiation of macrophages into the M2 phenotype so due to the widespread expression of SMP receptors facilitate resolution.

### 5.3 Receptor expression responsible for DAMP-RAMP transition on M2 cells

Over time, the signaling pathways that support inflammatory processes may weaken and those that promote dissolution and regeneration become more potent. The microenvironmental conditions promote the differentiation of M2 macrophages, while the functions of the M2 cells favor the resolution processes. This positive feedback accelerates the transition once the inflammation-resolution balance is tipped over (93). An important part of this regulation is that the receptors that convert DAMP signals into resolution signals are primarily expressed in M2 cells.

CD24 is considered a “don’t eat me” signal molecule. Siglec-10, the receptor for CD24, is expressed at a higher level in the M2 subtypes (the highest level in the M2c) (144). CD24 can be in complex with HMGB1 and induces the activation of Siglec-10, leading to anti-inflammatory signal transduction (145). By cooperating with CD24, Siglec-10 can also transform the inflammatory potential of other DAMPs, such as HSP70 or HSP90, into resolution (145). Similarly, HMGB1 loses its inflammatory potential in the presence of C1q. The C1q-HMGB1 complex activates the LAIR receptor of efferocytes, which inhibits inflammatory signals (146, 147). Like Siglec-10, LAIR1 expression is higher in M2 than in M1 macrophages (148). Thus, the engulfment of cellular debris by M2, but not by M1 cells, turns the effects of DAMPs into anti-inflammatory.

The expression of CD39 and CD73, the receptors responsible for the ATP-adenosine transition, is higher in the M2 than in the M1 phenotype based on published data (149). In contrast, some transcriptomic studies showed that the expression of CD73 was higher in M1 cells. Functionally, the hydrolysis of ATP to adenosine is more intense in M2 than in M1 cells (149). The generated adenosine inhibits the functions of M1 cells through A2A receptors and induces the polarization and activation of M2 cells through A2B receptors (70, 150). The ATP-P2X7Rs axis plays a role in inflammation in M1 cells, P2X7Rs might play an anti-inflammatory role in M2 macrophages (151). In intermediate M1/M2-polarized macrophages, extracellular ATP acts through its pyrophosphate chains, to inhibit IL-1β release (151), while P2X7R signaling does not induce NLRP3 activation in M2 macrophages (71).

The expression of the IL-33 receptor, ST2, is increased on M2 cells (128). In addition to the fact that IL-33 commonly induces M2 differentiation, IL-33 released from dying cells, or produced even by M2 cells (152), increases the expression of CD73, CD39 and MerTK, promoting other actors in tissue resolution (1).

PGE2 is known to have both a pro- and anti-inflammatory role. PGE2 binds to four different PGE2 receptors (EPs), of which EP4 mediates resolution signals. EP2 and EP4 receptors were detected on macrophages, but EP1 and EP3 receptors are barely or not expressed on these cells at all. LPS stimuli increase EP2 expression, thus modifying the EP2/EP4 ratio (130); however, increased expression of EP4 has also been demonstrated in M1 cells (131). In addition to SPMs,

Formyl peptide receptors (FPR) also recognize ANXA-1. FPR1 is mostly proinflammatory, while FPR2 has a pro-resolution effect. As mentioned, FPR2 expression is higher on M2 cells (133), moreover, IL-4 and IL-13 downregulate FPR1 expression on alternatively activated macrophages, providing a rather anti-inflammatory response to ANXA-1 on M2 cells (153). While the role of ANXA1 in promoting M2 differentiation is clear, further studies are needed to determine the ratio of FRP1/FRP2 receptors expressed on M1 and M2 cells and to demonstrate the potential differences in ANXA-mediated signaling in the two cell subtypes.

Overall, M2 macrophages, by expressing certain targeted receptors, have a high potential to convert the inflammation-inducing potential of DAMP molecules into an anti-inflammatory “RAMP-like” effect. After functional conversion, these DAMPs promote M2 differentiation, thereby accelerating the resolution process.

## 6 Comparison of RAMP- and DAMP-induced signaling pathways on polarized M1 and M2 cells

The pattern of DAMPs/RAMPs/SPMs released during sterile inflammation, depending on the different forms of cell death (1), results in the activation of many different receptors that can generate various stimuli. In the next chapter, we focus on the signaling initiated by DAMP/RAMP/SPM receptor ligation and the transcription factors activated.

As reviewed by Igor Malyshev et al. (154), there are two types of macrophage reprogramming signaling pathways: pathways that mainly program the M1 phenotype through the activity of Notch, TLR/NF-κB (р65/р50), PI3K/Akt2, JAK/STAT1, and HIF1α, as well as those pathways which mainly program the M2 phenotype through PI3K/Akt1, JAK/STAT3/6, TGFβ/SMAD, TLR/NF-κB (р50/р50), and HIF2α (154). Briefly, the profile of pro-inflammatory stimuli induces the polarization of M1 cells *via* the activation of transcription factors such as NF-κB, STAT1, STAT3, AP-1, SREBP-1, and HIF1α, while anti-inflammatory M2 macrophages are mainly controlled by transcription factors STAT6, GATA3, PPARγ, C/EBPββ, SP1, cMyc and LRX (155).

### 6.1 DAMP signal for M1-M2 differentiation and/or functions

**Figure 3:**
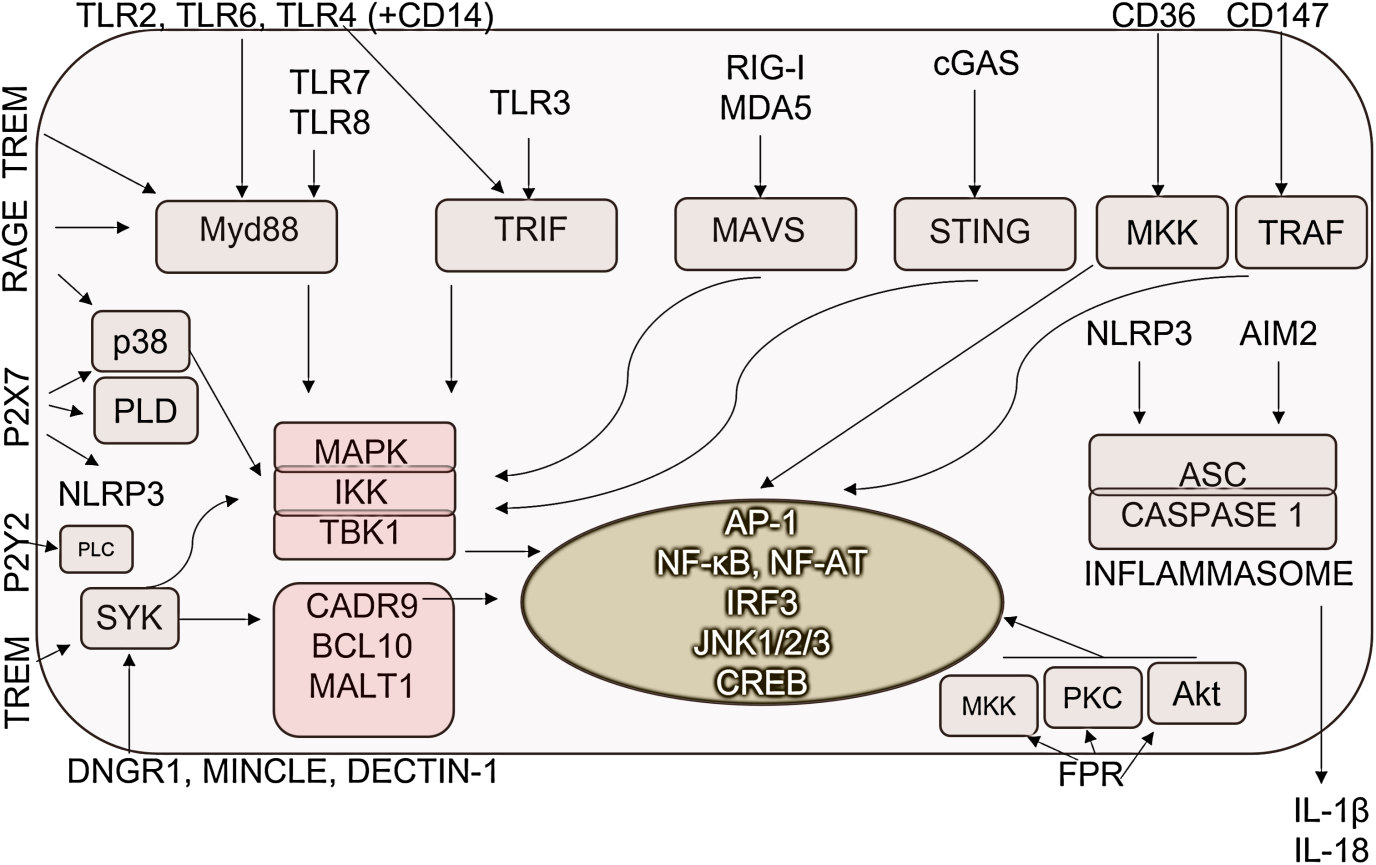
DAMP-sensing receptor-induced signaling pathways. DAMPs are recognized by pathogen-recognition receptors as well as various receptors specialized for the detection of human molecules. Their detection dominantly resulted in the activation of inflammation-promoting signaling pathways and transcription factors. AP-1, Activator protein 1; ASC, Apoptosis-associated speck-like protein containing a CARD; CD, cluster of differentiation; CREB, Cyclic AMP,Responsive Element-Binding Protein; DNGR-1, dendritic cell NK lectin group receptor-1; IKK, IκB kinase; JNKs- c,Jun N-terminal kinases; MAPKs, Mitogen-activated protein kinases; MALT1, mucosa-associated-lymphoid-tissue lymphoma-translocation gene 1; MAVS, Mitochondrial antiviral-signaling protein; MDA5, melanoma differentiation-associated protein 5; Mincle, Macrophage Inducible C-Type Lectin; MKK, mitogen-activated protein kinase kinase; MYD88, Myeloid differentiation primary response 88; NF-AT, Nuclear factor of activated T-cells; NF-κB, Nuclear factor-κB; NLRP3, NLR family pyrin domain containing 3; PKC, protein kinase C; PLC, Phospholipase C; PLD, Phospholipase D; RAGE, Receptor for Advanced Glycation Endproducts; RIG-I, retinoic acid-inducible gene I; STING, Stimulator of interferon genes; SYK, Spleen tyrosine kinase; TBK1, TANK-binding kinase 1; TLR, Toll-like receptor; TRAF, TNF receptor-associated factor; TREM1/2, Triggering receptor expressed on myeloid cells ½; TRIF, TIR-domain-containing adapter-inducing interferon-β.

DAMP-recognizing PRRs could act in an additive or synergistic manner. TLRs can activate MyD88-IKK-NF-κB, and MyD88-MAPK-AP1 pathways, in addition, TLR3 and TLR4 signaling results in the TRIF-TBK1-IRF3/7 activation. Accordingly, DAMP-sensing receptors; TLR2, TLR3, TLR4 and TLR7 enhance the activity of AP-1 (156–158). TLRs, like TLR3, TLR4 and TLR9 induce the activation of M1-associated IRF3 and IRF5 transcription factors in macrophages (156, 159). Additionally, C-type lectin receptors through activating SYK kinase stimulate the MAPKs (AP1) and NF-κB pathways (160). NOD-like receptors with the help of adapter protein apoptosis-associated speck-like protein containing CARD, also PYCARD (ASC) activate inflammasome for caspase-1 activation resulting IL-1β and IL-18 release (161) (**Figure 3**).

Receptors specialized to recognize exclusively DAMPs also activate signaling pathways in macrophages. IL-1 induces similar signaling through the IL-1R as TLRs activating MyD88/IKK complex leading to NF-κB and MAPK activation. RAGE receptor activates multiple signaling pathways, such as NF-κB p65, MAP kinases, RAC1, PI3K, SMAD and the Wnt-β pathway (162, 163). M1-expressed STAT1/5 also can be triggered by RAGE and FPR2 (164, 165). TREM receptors activate MAPK-AP1 pathways. In addition, TREM1 receptors, which are mainly expressed on M1 cells, stimulate NF-κB (166), while TREM2, which is expressed on M2 cells, activates PI3K *via* DAP10 mediating anti-inflammatory signaling (167). Syk-mediated signaling increased Ca^2+^ concentration, thus contributing to M2-M1 macrophage polarization by activating NFAT (168, 169). MINCLE activates the HIF-1α transcription factor (170).

Focusing on M2-related signaling, TLR4 and MINCLE also activate the M2-specific transcription factor C/EBPββ (170), while VEGFR- and CD147-derived signals resulted in the activation of M2-associated SP1 (171, 172). TLR activation can result in the generation of p50/p50, NF-κB homodimers without transcriptional activity, determining macrophage polarization (93).

A more precise understanding of differentiation would require a description of the fine regulation of signaling pathways in macrophages upon DAMP stimuli. PI3K/Akt pathway could also be induced by DAMPs and has been generally considered as a negative regulator of TLR and NF-κB signaling in macrophages, resulting in M2 polarization (173).. However, each isoform of PI3K and the activated AKT can explain the dual role of this pathway in macrophage polarization (35). The switching between the Akt1-Akt2 is responsible for the macrophage plasticity, Akt1 promotes M2 and Akt2 promotes M1 phenotype formation (35). This process is regulated by miR-155 (174). In acute inflammation, the “positive signals”, namely, NF-κB, ERK, miR-155 and PTEN repress the inhibitory signals of PI3K/Akt/PDK-1 pathway, while during resolution the PI3K/Akt1 pathway excess initiates the activation/increased production of C/EBPββ, SOCS1, IRAK-M, miR-146a and IL-10 associated with M2 polarization (173).

Priming of cells can deterministically modify the outcome of signaling. The increased activity of STAT1 in the IFN-γ-reprogrammed macrophages produces a more intensive response to TLR4 ligands (175). IFN-γ and the TLR4 ligands activate the synthesis of SOCS3, which blocks STAT3, thereby preventing the formation of the M2 phenotype, but IL-4 activates the synthesis of SOCS1, which blocks STAT1, thereby preventing the generation of M1 cells (176).

Overall, DAMP sensing appears to predominantly, however not exclusively, reprogram macrophages to the M1 phenotype. Both increased expression of PRRs on M1 cells and activated signaling pathways contribute to M1 differentiation.

### 6.2 RAMP signal for M1-M2 differentiation and/or functions

**Figure 4:**
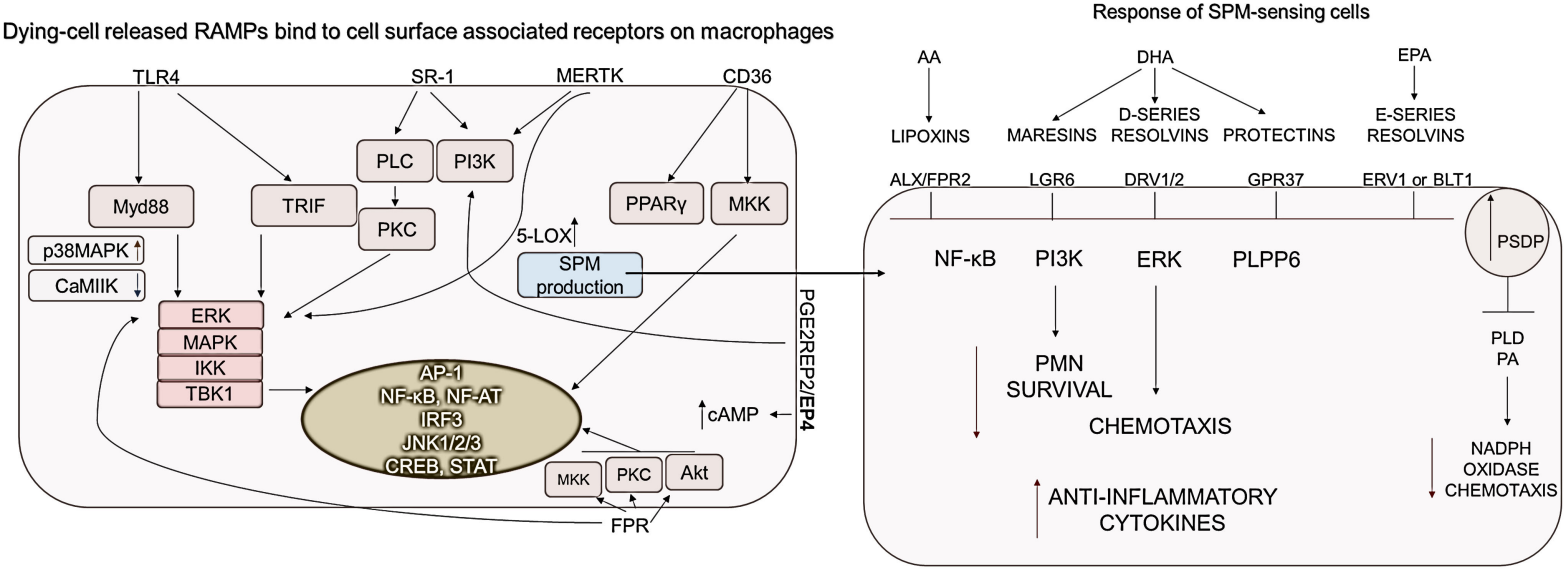
RAMP and SPM-sensing receptor-induced signaling pathways. Macrophages express receptors to sense RAMPs released from dying cells. CD36, PGE2R-derived signals, as well as MerTK activation, which detects phosphatidylserine on dying cells, are essential for increasing cAMP and SPM production. Accordingly to the DAMP/RAMP ratio and the generated signals, the SPMs decrease the survival of neutrophil granulocytes as well as their chemotaxis, while the production of anti-inflammatory cytokines is increased. ALX, Lipoxin receptor; BLT1, Leukotriene B4 receptor 1; CD, cluster of differentiation; DRV1, D-resolvin receptor 1; ERK, Extracellular signal-related kinase; ERV1, human resolvin E1 receptor; FPR, Formyl peptide receptor; GPR37, G Protein-Coupled Receptor 37; IKK, IκB kinase; JNKs- c,Jun N-terminal kinases; LGR6, Leucine-rich repeat-containing G protein-coupled receptor 6; 5-LOX, Expression of 5-lipoxygenase; MAPKs, Mitogen-activated protein kinases; MerTK, MER proto-oncogene, tyrosine kinase; MKK, mitogen-activated protein kinase kinase; MYD88, Myeloid differentiation primary response 88; NF-AT, Nuclear factor of activated T-cells; NF-κB, Nuclear factor-κB; PI3K, phosphoinositide-3 kinase; PKC, protein kinase C; PLC, Phospholipase C; PLD, Phospholipase D; PLPP6, phospholipid phosphatase 6; PPARs, peroxisome proliferator-activated receptors; SPM, Specialized pro-resolving mediators; SR-A1, Class A1 scavenger receptors; TBK1, TANK-binding kinase 1; TLR, Toll-like receptor; TRIF, TIR-domain-containing adapter-inducing interferon-β.

More RAMPs are likely to be released during necrotic cell death than during apoptosis, similar to what has been observed for DAMPs (reviewed in (1)). Released RAMPs act simultaneously with DAMPs to initiate the silencing of DAMP-induced signaling (177, 178) (**Figure 4**).

TLR4, SR-A, CD36, PGE2R EP2/EP4, MERTK are responsible for sensing the presence of HSP10, HSP27, αβ-crystallin, BiP, AnnexinA1 RAMPs (92, 115, 118, 135, 179–181). Signal transduction induced by extracellular HSPs is poorly understood. Hsp27 stimulation activates the ERK, c-Jun, and p38 MAPK pathways, of which only p38 was required for monocyte IL-10 production (182). Similarly, BiP-induced IL-10 production was reduced by the addition of the MAPK p38 pathway inhibitor SB203580, but unaffected by the ERK-1/2 inhibitor PD98059 (183, 184). This is supported by the fact that, among the MAPKs, p38 MAPK appears to play a dominant role in IL-4-induced alternative activation of macrophages (185). CD36 stimulation by HSP proteins results in PPARγ activation, indicating that high CD36 expression can convert the function of HSP proteins described as DAMPs into resolution-promoting activity. Although CD36 is rather expressed by M2 cells, it has been mentioned to induce the activation of STAT1/5 leading to the M1 cell polarization (138).

Clearance of dying cells by efferocytosis initiates a significant phenotypic shift within macrophages towards an anti-inflammatory, IL-10 and TGFβ producing phenotype. The activation of the most important receptor of efferocytosis, the MerTK receptor, either by Gas6, protein S or PS activates the MAPK, AKT, STAT6, pathways in macrophages, which results in M2 polarization (186, 187). In addition, MerTK reduces cytoplasmic calcium levels, thereby initiating two important pro-resolving processes (1): suppressed calcium levels reduce CaMKII activity, which results in MerTK synthesis, thus amplifying the pro-resolving program (188), and (2) suppressed CaMKII silences the MK2 activity which upregulates non-phosphorylated form of 5-LOX. This promotes its translocation from the nucleus into the cytoplasm, mediating the synthesis of SPMs (189).

SPMs could be recognized by receptors: ALX/FPR2, ChemR23, GPR32, GPR37, RORα, DRV1/2, ERV1 or BLT1, and LGR6. The ligation of LXA4 to ALX/FPR2 transmembrane receptor, as the binding of DHA- or EPA-derived mediators to their receptors, acts as a “stop signal” during inflammation by inhibiting NF-κB and ERK pathways among others (190)(**Figure 4**).

Changes in the cAMP levels are determined during the DAMP/RAMP/SPM-regulated resolution. Most SPMs, such as Resolvin D1, Resolvin D2, and N-3 docosapentaenoic acid–derived Resolvin D5, LXA4, maresin-1, increase intracellular levels of cAMP. In contrast, Resolvin E1 reduces cAMP levels *via* the activation of ERV-1/ChemR23 (191). The production of cAMP induces the activation of Epac1/2 and PKA signaling stimulating the p38-MAPK pathway, which results in the CREB activation. Consequently, PKA inhibits the GSK3, PI3K/Akt and NF-κB pathways (191). Stimulated Epac1/2 promotes the activation of SOCS3 *via* the stimulation of RAP1, c-Jun, and C/EBPβ (192). At the same time, cAMP-stimulated Epac1/2 also inhibits the NF-κB- and GSK3β-mediated pro-inflammatory cytokine production (193). In addition, an increase in cAMP level can promote IL-4-dependent M2 marker expression through a PKA/C/EBPββ/CREB-dependent pathway in murine macrophages (194). SPMs stimulate resolution in a positive feedback loop by activating the phosphorylation of 5-lipoxygenase (5-LOX), leading to further SPM production (191). SPMs (via DRV1/2, BLT1) dominantly induce the polarization of M2 cells after the activation of C/EBPββ and SP1 transcription factors (195, 196).

Macrophage phenotype switching has been proposed as an important step in the transition of a pro-inflammatory reaction into the resolution phase. The alterations of macrophage phenotypes are regulated by lipid mediators switching from pro-inflammatory lipid mediators such as leukotrienes and prostaglandins to SPMs (197). In addition to increased 5-LOX expression upon SPM stimuli, 12-LOX (198) and 15-LOX (178) expression levels are also elevated in M2 cells, and these enzymes are upregulated by SP1 and STAT6- dependent manner (199, 200).

Importantly, the levels of SPMs are much lower than typical pro-inflammatory mediators including the monohydroxylated fatty acid derivatives, leukotrienes, or certain prostaglandins. In the majority of the studies, the concentration is low (<50 pg/ml) which is close to the detection limit of several methods or below (178), drawing attention to the importance of further investigations.

In summary, SPMs typically promote alternative macrophage polarization, but the fact, that M1 cells could be also primed by these receptors, suggests the existence of M1 (associated with inflammatory or resolution-promoting) subpopulations similar to M2, again emphasizing that the functions of the macrophage populations are not sharply separated, in contrast to the M1 M2 classification. Overall RAMP signaling completed with SPM-induced changes is generally characterized by activation of cAMP and phosphorylation of p38 resulting in the M2-related transcription and a dominant inhibition of the NF-κB pathway to block macrophage polarization to M1 cells.

### 6.3 Modification of signaling pathways by DAMP-RAMP transition

Extracellular HMGB1 can also activate TLR4 and RAGE receptors. While disulfide HMGB1 mostly stimulates TLR4, similar to LPS, resulting in inflammatory responses, sulfonyl HMGB1, on the other hand, leads to an anti-inflammatory response through RAGE (69).. The presence of soluble C1q or the “don’t eat me” signals remaining on the surface of necrotizing cells causes co-stimulation of Siglec-10 or LAIR receptors with HMGB1-mediated signaling (69). Both receptors contain an ITIM motif inducing the activation of SHP-1 and SHP2 phosphatases blocking both NF-κβ and IRF signaling and terminating the inflammatory effect of HMGB1 (201, 202).

ATP binding to P2X7R results in NLRP3 inflammasome activation and the elevated intracellular Ca^2+^ concentration leads to MAPK and calcineurin (NFAT) activation favoring M1 differentiation (203). In contrast, adenosine in macrophages through A2A and A2B receptors increases intracellular cAMP levels resulting in CREB and STAT3 stimulation and simultaneous inhibition of NF-κB (150). In addition, *via* phosphorylation of p38, activated SOCS3 up-regulates IL-10 production and induces VEGF production while phosphorylating STAT3 promotes the polarization into M2 phenotype (191, 204).

IL-33, as a member of the IL-1 family, binds to the ST2 (ILRL1) receptor and activates MyD88- AP1, NF-κB signaling through the intracellular Toll/IL-1 receptor (TIR) domain characteristic of the receptor family (205). Its role in resolution is primarily due to the fact that the ST2 receptor is dominantly found on the surface of mast cells, type 2 innate lymphoid cells (ILC2), Th2 and regulatory T cells, the activation of which results in the IL-4, IL-13 signal (73). However, the ST2 also stimulates the GATA3 transcription factor (158) and IL-33 inactivates GSK-3β through an ST2-independent pathway (206).

Among the PGE2 receptors, EP2 and EP4 receptor stimulation increases the intracellular cAMP level (207). EP4 is also able to induce tolerogenic signals *via* activating the phosphatidylinositol 3-kinase pathway and activating C/EBPββ which is an M2-associated transcription factor (130). PGE2 stimulation also enhances M2 polarization through Krupple-like factor 4 (KLF4), CREB and GATA3 factors (75, 208). As the production of PGE2 is increased as a result of the induction of cell death, it is an important negative regulator of the immune response during sterile inflammation (209).

As ANXA1 can also induce a proinflammatory signal, recombinant ANXA-1 promotes myeloid differentiation by activating the ERK1/2-NFAT2 pathway and increasing intracellular Ca^2+^ concentration (210). Although ANXA-1 binds to the same FRP2 receptor as LXA4, the effect of ANXA1 on directly increasing cAMP levels is still uncertain (191). Conversely, the elevated cAMP level increases ANXA-1 expression and activity, which promotes the efferocytosis of apoptotic leukocytes by macrophages (191). ANXA1 increases the expression of PPARγ by regulating the phosphorylation of STAT6 and as positive feedback, PPARγ upregulates the expression of ANXA1 (211).

## 7 Limitations in the field/knowing the limit

Although the significance of sterile inflammation is considerable, most inflammation studies primarily focus on pathogen responses. Due to limited research, the phases of sterile inflammation, including cell death, DAMP release, inflammation, and resolution, have not been effectively interconnected. Some articles explore cell death and DAMP release, while others examine the role of macrophages in inflammation and resolution. However, the impact of cell death and DAMPs on macrophages during sterile inflammation remains poorly investigated. Numerous *in vivo* models induce sterile inflammation and muscle regeneration, but they often overlook the processes of cell death. Although various cell death pathways are well characterized, their immunological outcomes and especially their effects on resolution are inadequately studied. Further research is needed to establish connections between these isolated models.

The usage of M1 and M2 nomenclature is widespread. Cells obtained through different differentiation protocols are uniformly designated as M1 or M2, often without considering subpopulations. *In vivo*, macrophage subtypes exhibit greater complexity than the oversimplified M1/M2 classification based on *in vitro* studies. Within tissues, macrophage subsets exist on a continuum and are regulated differently based on the tissue type. Our review highlights the significant differences between DAMP-induced inflammation/resolution and PAMP-induced inflammation, which has been understudied thus far. This suggests the presence of distinct macrophage subpopulations and functionality, including various transitional forms.

## 8 Conclusions

The regulation of the transition between inflammation and resolution remains incompletely understood. The conversion of pro-inflammatory macrophages into resolution-inducing cells is a pivotal step in this process. Signaling mediated by resolution-promoting cells triggers positive feedback loops that determine the final outcome. Therefore, identifying the regulators of this tipping point holds great therapeutic potential. While M1 and M2 cells share certain functional elements, such as the expression of pattern recognition receptors (PRRs) and associated signaling pathways, more notable differences arise in their response to damage-associated molecular patterns (DAMPs) and resolution-associated molecular patterns (RAMPs) compared to pathogen-associated molecular patterns (PAMPs). Targeting the RAMP-DAMP pathway for optimal therapeutic intervention can pave new paths in inflammation and resolution regulation. Understanding the role of DAMPs and RAMPs released from dying cells during sterile inflammation is imperative. In-depth investigation using *in vitro* and, more importantly, complex *in vivo* models specifically designed to study sterile inflammation and its interdependent phases of cell death, DAMP release, and macrophage polarization is required. Exploring critical regulatory points and molecular mechanisms in the transition, such as HMGB1 and ATP conversion to an anti-inflammatory phenotype, as well as differences in AKT1/AKT2 and TREM1/TREM2 signaling, may offer insights to control sterile inflammatory pathological conditions effectively.

## Author contributions

Conceptualization, AM, and GK. Writing—Original Draft Preparation, GK, AM, VJ, EV, MT, and BK. Writing—Review and Editing, GK, AB, VJ, MT and AM. Supervision, AM. Funding Acquisition, AM, VJ and GK. All authors contributed to the article and approved the submitted version.
